# Validity and Reliability of the Integrated Palliative Care Outcome Scale in Asian Heart Failure Patients

**DOI:** 10.1089/pmr.2022.0029

**Published:** 2022-11-21

**Authors:** Shirlyn Hui-Shan Neo, Jasmine Yun-Ting Tan, David Kheng-Leng Sim, Elaine Swee-Ling Ng, Julian Kenrick Xingyuan Loh, Grace Meijuan Yang, Fliss E.M. Murtagh, Yin Bun Cheung

**Affiliations:** ^1^ Division of Supportive and Palliative Care, National Cancer Centre Singapore, Singapore, Singapore.; ^2^ Lien Centre for Palliative Care, Duke-NUS Medical School, Singapore, Singapore.; ^3^ Department of Cardiology, National Heart Centre Singapore, Singapore, Singapore.; ^4^ Nursing Specialty Care Unit, National Heart Centre Singapore, Singapore, Singapore.; ^5^ Wolfson Palliative Care Research Centre, Hull York Medical School, University of Hull, Hull, United Kingdom.; ^6^ Program in Health Services & Systems Research and Centre for Quantitative Medicine, Duke-NUS Medical School, Singapore, Singapore.; ^7^ Centre for Child, Adolescent and Maternal Health Research, Tampere University, Tampere, Finland.

**Keywords:** health care staff, heart failure, integrated-palliative-care-outcome-scale, patients, reliability, validity

## Abstract

**Background:**

The Integrated Palliative Care Outcome Scale (IPOS) was developed in the United Kingdom for health assessment in advanced illness.

**Objectives:**

To evaluate the validity and reliability of a culturally adapted IPOS (both patient and staff versions) for heart failure (HF).

**Design/Setting:**

We recruited HF patients and staff from a tertiary hospital in Singapore. We collected patient IPOS, New York Heart Association (NYHA) status, Edmonton Symptom Assessment System (ESAS) and Minnesota Living with Heart Failure (MLHF) scores at baseline, and patient IPOS at follow-up. Each baseline patient IPOS was matched with a staff IPOS.

**Measurements:**

Pearson correlation coefficient (*r*) between ESAS, MLHF, and patient IPOS was calculated to assess construct validity. The two-sample *T*-test assessed difference in patient and staff IPOS scores across NYHA status and care settings for known-group validity. Internal consistency of patient and staff IPOS was assessed using Cronbach's alpha (*α*). Intraclass correlation coefficient (ICC) was used to assess test-retest reliability of patient IPOS and inter-rater reliability between patient and staff IPOS.

**Results:**

Ninety-one patients and 12 staff participated. There was strong convergent validity of total patient IPOS with MLHF (*r* = 0.78) and ESAS (*r* = 0.81). There were statistically significant differences in total IPOS across care settings (patient-IPOS: 8.05, staff-IPOS 13.61) and NYHA (patient-IPOS: 7.52, staff-IPOS 12.71).

There was high internal consistency of total patient (*α* = 0.83) and staff IPOS (*α* = 0.88) and high test-retest reliability of patient IPOS (ICC 0.81). Inter-rater reliability (ICC) ranged between 0.82 and 0.91.

**Conclusion:**

The IPOS was valid and reliable for HF patients in Singapore.

## Introduction

The incidence of heart failure (HF) is projected to increase worldwide.^1^ Cardiovascular disease is also a significant cause of mortality and morbidity in Singapore and South-East Asia.^2,3^ End-stage HF is characterized by functional decline, physical and psycho-emotional distress, and financial stress.^4–6^ However, these problems are often under-reported by patients and under-identified by health care staff.^7–9^

Systematic screening of patients with HF, using patient- and staff-reported tools, is one possible way of identifying HF patients who have these needs. Patients can then be referred to palliative care (PC) specialists for support. PC support has been shown to reduce symptom burden and hospitalizations for patients with HF.^10–15^

The Integrated Palliative Care Outcome Scale (IPOS) was developed in the United Kingdom. It combines the Palliative Care Outcome Scale (POS) (which assesses psycho-emotional, practical, and informational needs) with a symptom module that assesses common symptoms in patients living with serious illnesses.^16^ Compared with other tools that are available for PC needs screening, the IPOS is brief, and it has good psychometric properties.^17^ In the United Kingdom, the regular use of IPOS during clinical visits helped improve HF patient self-reporting and staff identification of needs.^18,19^

Assessment tools developed in another country may not be entirely or readily usable in a new setting because of differing socio-economic-cultural contexts and disease-specific impacts.^20^ In an earlier study, the team culturally adapted the IPOS for cancer patients in Singapore.^21^ This was done through an iterative process of cognitive interviews with patients to adapt the IPOS items in accordance with existing guidelines.^22^

However, as patients with HF have a different prevalence of symptoms and needs compared to patients with cancer,^7^ this study aimed at evaluating the validity and reliability of the culturally adapted IPOS^21^ in patients with HF in Singapore.

## Methodology

### Study setting

This was a prospective study carried out in the inpatient and outpatient setting of the National Heart Centre Singapore, a large national and regional referral center for patients with cardiac diseases.^23^

### Validation process

#### Participants and recruitment process

The study team consisted of clinicians from a multidisciplinary HF and PC team. The research coordinator screened patients of the study team and patients who were referred to the study team for eligibility.^24^ Eligible participants were invited to participate, and those who consented were recruited. Patient participants were at least 21 years old, aware of their diagnosis, had a clinical diagnosis of HF as deemed by their managing cardiologists, and could speak English. English is the most common language spoken at home (48.3% of the population), as in the 2020 Singapore census.^25^

For staff participants, we recruited nursing and physician staff from the multidisciplinary PC and HF teams who directly cared for the recruited patient participants. Staff participants were not part of the study team.

### Data collection

#### Study measures

IPOS: The IPOS is reported by both patients and staff. The patient and staff IPOS are similar except for the last item (not presented in the staff version), which asks the patient if the tool was self-completed or completed with assistance. The first item “What have been your main problems or concerns over the past three days?” required participants to report their main problems and concerns in free text. The IPOS used in this study^21^ had an additional descriptor (health, financial, well-being etc.) for the first item, to provide participants with an additional context for answering this question. Apart from the first item, the other items in the IPOS capture patient needs and symptoms over the past three days.

The scores on all items are summed to form a total score, and a higher IPOS total score indicates more needs. The other subscales in the IPOS (physical symptoms, emotional symptoms, communication/practical issues) were derived by summing up the scores of the respective individual items in the respective subscales.^26^

The Edmonton Symptom Assessment System-revised (ESAS-r)^27^ assesses physical symptoms such as pain and emotional symptoms such as anxiety. The item scores for the ESAS can be summed, and a higher total ESAS-r score indicates worse symptoms. For this study, the items related to physical and emotional symptoms were summed to form physical and emotional subscales scores respectively.^28^

The Minnesota Living with Heart Failure (MLHF)^29^ questionnaire assesses physical and emotional needs that impact a patient's quality of life. The item scores on the MLHF can be summed, and a higher total score implies a worse quality of life. The MLHF has specific instructions for summing up physical and emotional items to form physical and emotional subscale scores, respectively.

#### Time points

Data were collected at baseline and follow-up. At baseline, we collected clinical diagnoses from the electronic health records, and patient demographics and New York Heart Association (NYHA) functional status via self-report. Patients filled in the IPOS, the ESAS-r and MLHF. ESAS-r, MLHF, care setting and NYHA status were used as validity criteria for evaluation of the patient IPOS. The primary health care staff caring for the patient was asked to fill in the staff IPOS for his/her patient, such that each patient's IPOS would be matched by a staff IPOS.

A follow-up patient IPOS was conducted within two weeks after baseline.^30^ Patients also answered a question related to change in main concerns and problems: “Since the questionnaire was last completed, thinking about your main problems and concerns, would you say that: things have got much better, things have got a little better, there has been no change, things have got a little worse, things have got much worse?.” This question was used to define eligibility for inclusion for test-retest reliability (those who reported “no change,” “a little better” or “a little worse” would be included).

### Statistical analysis plan

We described the percentage of participants who scored the lowest (floor) or highest (ceiling) possible scores to evaluate for any significant floor or ceiling effect (defined as ≥20%) for both the patient and staff IPOS.^31^ An overall IPOS score was not generated, if any items within the IPOS were marked “not assessed” or “missing.”^32^

To assess the construct validity (*overall validity*)^33^ of the patient IPOS, we measured the Pearson correlation coefficient (*r*) between the total IPOS scores and the summative scores of the ESAS and MLHF. We also correlated the IPOS subscale scores with the respective subscale scores of the ESAS and the MLHF. To assess known-group validity (*ability of IPOS to distinguish between groups*),^33^ we compared patient and staff IPOS scores in the (1) inpatient setting versus the outpatient setting and (2) those with non- to slight limitation of physical activity (NYHA score 1–2) versus those with marked limitation of physical activity or who were unable to carry out any activity (NYHA score 3–4); using the two-sample *t*-test.

We calculated Cronbach's alpha (*α*) to examine the internal consistency (*how well the IPOS items measure the same construct*)^33^ of the patient and staff IPOS. For test–retest reliability (the *degree to which IPOS assessments are consistent from one assessment to the next*),^33^ we calculated the intraclass correlation coefficient (ICC) between the patient IPOS scores at baseline and follow-up for participants who responded that there has been no or minimal change in their main problems and concerns. We also explored the ICC between the patient and staff IPOS across the total scores and subscales for inter-rater reliability (*degree of agreement between two raters*).^33^

### Sample size estimation

To establish convergent validity, we required a minimum sample size of 80 to provide 80% power, at 5% type 1 error rate, to test a correlation coefficient of 0.4 between the total score of IPOS and ESAS, against a trivial correlation of 0.1.^34^ To account for dropouts, at least 90 patients with HF were recruited.

### Ethics

This study was reviewed and approved by the SingHealth Centralized Institutional Review Board (Ref. No. 2019/2344). All participants provided written consent before participation.

## Results

### Participant characteristics

The demographic characteristics of patients and staff are presented in Table 1. We recruited 91 patients. Corresponding staff IPOS assessments were collected from 12 health care staff. There was a multi-racial distribution of patients, and the Chinese were the most common race (53.9%). The most common cause of HF was ischemic heart disease (56.0%). The mean number of years since HF diagnosis was 6.3 years.

**Table 1.** Demographics and Characteristics of Participants

Patient participants (*N* = 91)	Mean (SD)/frequency (%)^a^
Age, mean (SD)	56.5 (13.5)
Gender	
Male	67 (73.6)
Female	24 (26.4)
Race	
Chinese	49 (53.9)
Malay	17 (18.7)
Indian	22 (24.2)
Others	3 (3.3)
Marital status	
Married	60 (65.9)
Single	18 (19.8)
Divorced	7 (7.7)
Widowed	6 (6.6)
Education	
No formal education	1 (1.1)
Primary	6 (6.6)
Secondary	44 (48.4)
Post-secondary	40 (44.0)
Causes of HF	
Ischemic heart disease	51 (56.0)
Inflammatory musculopathy	3 (3.3)
Other musculopathy	26 (28.6)
Valvular	1 (1.1)
Arrythmia	2 (2.2)
Congenital	3 (3.3)
Endocrine	3 (3.3)
Drug	3 (3.3)
Others	1 (1.1)
No. of years since diagnosis	
Mean (SD)	6.3 (5.6)
New York Heart Association functional status (at point of recruitment)	
1	36 (40.0)
2	34 (37.8)
3	20 (22.2)
4	0 (0.00)
LVEF (at point of recruitment)	
Mean (SD)	27.5 (12.3)
Median (IQR)	25 (16)
Mode of administration	
Interviewer assisted	30 (33.0)
Self	60 (67.0)
Setting of administration at baseline	
Inpatient	42 (46.2)
Outpatient	49 (53.9)

Health care staff participants (*N* = 12)	Mean (SD)/frequency (%)^a^
Age	
Mean (SD)	35.4 (3.1)
Gender	
Male	4 (33.3)
Female	8 (66.7)
Designation	
Associate Consultant/Consultant	6 (50.0)
Senior Resident/Resident Physician	2 (16.7)
Nurses	4 (33.3)
Years of experience with HF patients	
Mean (SD)	5.4 (3.1)
Primary specialty	
Palliative care	2 (16.7)
HF	10 (83.3)

^a^ Mean and SD for continuous variables, frequency (*N*) and percent for categorical variables.

HF, heart failure; IQR, interquartile range; LVEF, left ventricular ejection fraction; SD, standard deviation.

There were nearly equal number of patients in the inpatient setting (46.2%) and outpatient setting (53.9%). More than half of the patients (67.0%) were able to self-administer the IPOS. The mean total ESAS score was 14.31, and the mean total MLHF was 30.90 for the patient participants. The mean age of staff was 35.4 years. There were eight physicians and four nurses. Staff had a mean of 5.4 years of experience working with HF patients.

### Description of characteristics of patient and staff IPOS

A descriptive summary of the distribution of the patient IPOS is presented in Table 2. There were no missing responses in the patient IPOS. There was a floor effect (reports of no problems at all) across all individual items (from 30.8% for weakness to 87.9% for vomiting), except for the item related to family anxiety. There was a floor effect for the subscale of communication/practical issues but not for the physical and emotional symptom subscales and total patient IPOS scores. There was no ceiling effect for the individual items, subscales, or total scores.

**Table 2.** Descriptive Summary of Integrated Palliative Care Outcome Scale, Minnesota Living with Heart Failure and Edmonton Symptom Assessment System Scores in Patients (*N* = 91)

Scale	Domain	Item description	Mean (SD)	% Floor	% Ceiling
IPOS	Physical	Pain	0.58 (1.04)	68.13	4.40
		Breathlessness	1.08 (1.20)	43.96	4.40
		Weakness	1.26(1.13)	30.77	3.30
		Nausea	0.45 (0.83)	72.53	0.00
		Vomiting	0.21 (0.64)	87.91	0.00
		Poor appetite	0.82 (1.06)	51.65	1.10
		Constipation	0.29 (0.76)	82.42	2.20
		Sore or dry mouth	0.70 (1.07)	60.44	3.30
		Drowsiness	0.38 (0.77)	74.73	0.00
		Poor Mobility	0.74 (0.99)	56.04	1.10
		Domain score	6.52 (5.42)	11.00	0.00
	Emotional	Anxious (patient)	1.13 (1.14)	35.16	4.40
		Anxious (family)	1.81 (1.29)	18.68	10.99
		Depressed	0.75 (0.97)	52.75	1.10
		Peace	1.18 (1.13)	31.87	6.59
		Domain score	4.87(3.62)	9.90	0.00
	Communication/practical	Sharing	1.14 (1.25)	42.86	6.59
		Having information	0.89 (1.07)	47.25	3.30
		Practical problems	1.03 (1.16)	45.05	4.40
		Domain Score	3.07(2.63)	25.30	0.00
	All Items	Total score	14.45 (9.23)	2.20	0.00

IPOS, Integrated Palliative Care Outcome Scale.

For the staff IPOS (described in Table 3), 87 out of 91 (95.6%) of assessments were performed by the HF staff. There were no missing responses except for the individual item for vomiting (1.1%). Gastrointestinal symptoms, drowsiness, mobility, and psychosocial needs were commonly scored by staff as “unable to assess.” There was a floor effect across most individual items, except for those related to family anxiety, peace, information and practical needs. There was a floor effect for the physical, and emotional symptom subscales but not for the communication/practical issues subscale and total staff IPOS. There was no ceiling effect for individual items, subscale scores, or the total staff IPOS.

**Table 3.** Descriptive Summary of Integrated Palliative Care Outcome Scale Staff Reported Scores, for *N* = 12 Staff Who Provided *N* = 91 Assessments

Scale	Domain	Item description	Mean (SD)	% scored as unable to access	% Floor	% Ceiling	% Missing
IPOS	Physical	Pain	0.26 (0.66)	7.69	76.92	0.00	0.00
		Breathlessness	0.88 (0.99)	1.10	47.25	1.10	0.00
		Weakness	0.83 (0.82)	10.99	37.36	0.00	0.00
		Nausea	0.13 (0.39)	27.47	63.74	0.00	0.00
		Vomiting	0.03 (0.25)	31.87	67.03	0.00	1.10
		Poor appetite	0.41 (0.64)	30.00	46.67	0.00	0.00
		Constipation	0.02 (0.13)	37.36	61.54	0.00	0.00
		Sore or dry mouth	0.13 (0.33)	38.46	53.85	0.00	0.00
		Drowsiness	0.02 (0.13)	36.26	62.64	0.00	0.00
		Poor Mobility	0.58 (0.88)	25.27	47.25	0.00	0.00
		Domain Score	2.79 (3.01)	—	30.77	0.00	—
	Emotional	Anxious (patient)	1.19 (1.04)	15.38	26.37	1.10	0.00
		Anxious (family)	1.42 (1.16)	45.05	15.38	2.20	0.00
		Depressed	0.47 (0.80)	19.78	54.95	0.00	0.00
		Peace	1.29 (0.89)	31.87	10.99	1.10	0.00
		Domain score	3.42 (3.11)	—	22.20	0.00	—
	Communication/Practical	Sharing	1.29 (0.86)	53.85	5.56	0.00	0.00
		Having information	0.92 (0.63)	16.48	17.58	0.00	0.00
		Practical problems addressed	1.07 (0.92)	35.56	14.44	3.30	0.00
		Domain score	2.35 (1.90)	—	16.50	0.00	—
	All Items	Total score	11.25 (7.35)	—	6.25	0.00	—

### Validity results

For the patient IPOS (described in Table 4), there was a strong correlation between the total IPOS score and the total MLHF score (*r* = 0.78) and total ESAS score (*r* = 0.81), providing evidence of convergent validity. The physical symptom subscale scores of the IPOS also correlated moderately with the physical symptom subscale scores of the MLHF (*r* = 0.63) and the physical symptom subscale scores of the ESAS (*r* = 0.73).

**Table 4.** Pearson Correlation Coefficients (*r*) of the Patient Rated Integrated Palliative Care Outcome Scale; Edmonton Symptom Assessment System; Minnesota Living with Heart Failure Scores at Baseline (*N* = 91)

Correlation estimates (r)	MLHF total	MLHF physical	MLHF psychological	ESAS total	ESAS physical	ESAS psychological
IPOS total	0.78	0.71	0.75	0.81	0.75	0.72
IPOS physical	0.61	0.63	0.48	0.71	0.73	0.55
IPOS emotional	0.65	0.55	0.70	0.67	0.58	0.64
IPOS communication/practical	0.57	0.45	0.67	0.44	0.33	0.50

All *p* values were ≤0.001.

ESAS, Edmonton Symptom Assessment System; MLHF, Minnesota Living with Heart Failure.

The IPOS emotional symptom subscale scores correlated moderately with the MLHF emotional symptom scores (*r* = 0.70) and the ESAS emotional symptoms subscale scores (*r* = 0.64). There was a moderate correlation between the IPOS communication/practical issues subscale scores and the MLHF emotional symptoms subscale scores (*r* = 0.67) but less so with the ESAS emotional symptom subscale scores (*r* = 0.50). The IPOS communication/practical issues subscale scores correlated poorly with ESAS physical symptom subscales scores (*r* = 0.33), providing evidence of divergent validity (*degree to which measures that are theoretically un-related are un-related*).

Inpatients had higher IPOS scores than outpatients, demonstrating the ability of the IPOS to distinguish between patient groups (*known group validity*) (Table 5). This difference was statistically significant for the total IPOS (*p* < 0.001), IPOS physical symptom subscale (*p* < 0.001), and IPOS emotional symptom subscale (*p* = 0.006). For the comparison of patient IPOS across NYHA strata, differences were also seen, with lower scores among patients with lower NYHA scores. This difference was statistically significant for the total IPOS (*p* = 0.001) and IPOS physical symptom subscale scores(*p* < 0.001).

**Table 5.** Known Group Validity Comparisons for Inpatient Versus Outpatient Setting and New York Heart Association Score

Patient	Inpatient (*N* = 42)	Outpatient (*N* = 49)	Difference in means	*p*	Staff	Inpatient (*N* = 42)	Outpatient (*N* = 49)	Difference in means	*p*
IPOS total	18.79 (8.89)	10.73 (7.87)	8.05	*p* < 0.001	IPOS total	15.36 (1.37)	1.75 (0.63)	13.61	*p* < 0.001
IPOS physical	9.36 (5.73)	4.08 (3.71)	5.28	*p* < 0.001	IPOS physical	4.36 (3.32)	1.40 (1.87)	2.96	*p* < 0.001
IPOS emotional	5.98 (3.59)	3.92 (3.40)	2.06	*p* = 0.006	IPOS emotional	4.31 (3.20)	2.46 (2.74)	1.89	*p* = 0.007
IPOS communication/practical	3.45 (2.76)	2.73 (2.49)	0.72	*p* = 0.20	IPOS communication/practical	2.92 (2.13)	1.78 (1.42)	1.12	*p* = 0.006

Patient	NYHA (1–2) (*N* = 70)	NYHA (3–4) (*N* = 20)	Difference in means	*p*	Staff	NYHA (1–2) (*N* = 70)	NYHA (3–4) (*N* = 20)	Difference in means	*p*
IPOS total	12.69 (8.97)	20.20 (7.87)	7.52	*p* = 0.001	IPOS total	4.17 (1.66)	16.88 (1.33)	12.71	*p* < 0.001
IPOS physical	5.23 (4.82)	10.8 (5.31)	5.57	*p* < 0.001	IPOS physical	2.06 (2.39)	5.25 (3.73)	3.19	*p* < 0.001
IPOS emotional	4.56 (3.74)	5.95 (3.10)	1.39	*p* = 0.132	IPOS emotional	2.93 (3.02)	4.55 (2.86)	1.62	*p* = 0.039
IPOS communication/practical	2.90 (2.54)	3.45 (2.86)	0.55	*p* = 0.409	IPOS communication/practical	2.17 (1.89)	2.85 (1.90)	0.68	*p* = 0.172

NYHA, New York Heart Association.

For the comparison of staff IPOS across care settings, inpatients had higher IPOS scores than outpatients. This was statistically significant for the total staff IPOS, physical and emotional symptoms subscales, and communication/practical issues subscales. In the comparison of staff IPOS across NYHA strata, patients with lower NYHA scores had lower IPOS scores, and this was statistically significant for the IPOS total (*p* < 0.001), physical symptom subscale (*p* < 0.001), and emotional symptom subscale (*p* = 0.039).

### Reliability results

The values are presented in Table 6. For the patient IPOS, the total IPOS and emotional subscale of the IPOS demonstrated high internal consistency (*α* = 0.83 and 0.81, respectively) but α was lower for the physical (*α* = 0.76) symptoms and communication/practical issues subscales (*α* = 0.62). For the staff IPOS, the internal consistency was high for the physical symptom subscale (*α* = 0.93), total IPOS (*α* = 0.88), and emotional (*α* = 0.82) symptom subscales but lower for the communication/practical issues subscale (*α* = 0.71).

**Table 6.** Test–Retest Reliability of Patient Integrated Palliative Care Outcome Scale and Inter-Rater Reliability of Patient and Staff Integrated Palliative Care Outcome Scale

Characteristics of IPOS	Intra-class coefficient (test–retest reliability) (*N* = 40)	95% Confidence interval	Intra-class coefficient (patient vs. staff IPOS) (*N* = 15)^a^	95% Confidence interval
Physical	0.82	0.65–0.90^b^	0.84	0.70–0.94^b^
Emotional	0.83	0.68–0.91^b^	0.85	0.71–0.94^b^
Communication/practical	0.85	0.72–0.92^b^	0.82	0.64–0.93^b^
Total	0.81	0.61–0.91^b^	0.91	0.83–0.96^b^

^a^ Out of 91 IPOS questionnaires done by patient and staff at baseline, 15 staff-patient assessments were used to evaluate inter-rater reliability between patient and staff.

^b^ Indicates *p*-values were statistically significant (<0.05).

The mean interval between patient baseline and follow-ups was 4.27 days. The number of patients who participated in follow-up was 71, of whom 22 reported “no change” and 18 reported that their condition was either “a little better” or “a little worse.” For test–retest reliability among the patients who reported “no change,” “a little better” or “a little worse,” the ICC was high (0.81 for the total IPOS) and 0.82–0.85 across the subscales. Of the 91 staff assessments, 15 were complete and were used for comparison with the patient IPOS for inter-rater reliability. The ICC for inter-rater reliability for the patient IPOS against the staff IPOS was strong, ranging from 0.82 (communication/practical) issues to 0.91 (total IPOS score).

## Discussion

It is important to validate patient-and-staff-reported outcome tools across different cultures and disease populations. This is the first study to assess the measurement properties of the IPOS in patients with HF in Singapore. Our main findings were, first, that the patient and staff IPOS had no ceiling effects but significant floor effects for the individual items. Second, there was evidence of known group validity for both the patient and staff IPOS, and convergent and divergent validity of the patient IPOS. Third, the patient and staff IPOS had good internal consistency. Finally, the patient IPOS had good test–retest reliability.

The low ceiling and high floor effect of the IPOS are advantageous for its use as a screening tool. The use of IPOS as a screening tool was also demonstrated in a cohort of patients with HF in Germany where investigators successfully deployed the IPOS to screen for symptoms and found that clinically relevant symptoms were frequently reported in a significant proportion (75%) of patients.^35^

With respect to the inter-rater reliability of the IPOS, the number of complete staff responses that could be used for the analysis was less than expected. This could be because most of the staff assessments were conducted by non-PC staff. We postulate that they had documented that they could not assess a fair number of needs on the IPOS either due to a lack of time in the local clinical setting, or that it is generally deemed more important to evaluate physical needs such as breathlessness.

Of note, standard clinical guidelines for cardiologists emphasize the assessment of congestion status, rather than non-cardiac or emotional symptoms.^36^ A study with health care professionals working with HF patients in the Netherlands also showed that non-PC staff had limited time and were less comfortable in identifying PC needs.^37^ Further studies should be performed in the future, to understand the reasons for the lack of complete assessments by non-PC staff, and to design strategies that can be used to improve generalist PC education among non-PC staff and to support them with the assessment of the broad PC needs of HF patients. This could improve the utility of the staff IPOS locally for non-PC trained staff.

One of the strengths of this study is that we recruited both patients and staff, and we evaluated the IPOS in a multidisciplinary manner. Second, patients were recruited in a non-PC setting, in contrast to other studies where patients were recruited predominantly from PC services.^26^ Our findings are unique, as they shed light on how the IPOS could be used to screen the needs of HF patients in a non-PC setting. Lastly, the sample size was also sufficient, ensuring that the study was adequately powered.

The limitations of this study were as follows: First, the IPOS was validated in English, predominately male, and higher educated HF patients. However, this is alike the epidemiology of HF in Asian patients with HF where there is a predominant male population.^38^ The literacy profile of Singaporeans has also improved over time.^25^ More Singaporeans have attained post-secondary or higher qualifications and English is now most frequently spoken at home.^25^ There is value in the validation of tools across settings, diseases, and cultures,^39^ and future studies could consider the evaluation of IPOS in a more diverse population, including women, non-English speaking, and participants from lower socio-economic statuses.

Second, as we recruited consecutive patients who met the eligibility criteria and consented to participate, sampling bias may have occurred. For example, we did not manage to recruit NYHA class 4 patients. It is possible that they were too symptomatic and not referred for eligibility screening. However, we managed to recruit a sizeable number of class 3 patients, who have been shown to have similar exercise characteristics to class 4 patients.^40^

A study using the IPOS in the Japanese population also concluded that NYHA class 3 and 4 patients had similar needs, although worse functional status was associated with more severe symptoms.^41^ We believe that the IPOS is likely to be valid and reliable in NYHA class 4 patients.

Third, older patients with HF likely have a different spectrum of needs than patients with HF.^42,43^ However, this study did not have a pre-planned aim to describe the differences in IPOS scores across ages. Further studies should be performed to describe age-specific differences in the needs measured by the IPOS.

Finally, we used the three-day recall period for both inpatient and outpatient settings, as these are similar populations in our model of care. Further studies could be conducted in the local outpatient setting, to understand if the three-or-seven-day recall period would be more appropriate.

In conclusion, the patient and staff IPOS have acceptable validity and reliability in English speaking patients with HF, aged ≥21 years. These findings are alike IPOS validation studies in other settings and disease populations,^21,26,44–49^ and they support the use of IPOS for screening PC needs of local patients with HF.

## Abbreviations Used

ESAS: Edmonton Symptom Assessment System
ESAS-r: Edmonton Symptom Assessment System-revised
HF: heart failure
ICC: intraclass correlation coefficient
IPOS: Integrated Palliative Care Outcome Scale
IQR: interquartile range
MLHF: Minnesota Living with Heart Failure
NYHA: New York Heart Association
PC: palliative care
POS: Palliative Care Outcome Scale
SD: standard deviation
